# Effect of vitamin B12 on methotrexate-induced cardiotoxicity in rats 

**DOI:** 10.22038/IJBMS.2024.74161.16120

**Published:** 2024

**Authors:** Nurhan Kuloğlu, Derya Karabulut, Emin Kaymak, Ali Tuğrul Akin, Tayfun Ceylan, Ayşegül Burçin Yıldırım, Birkan Yakan

**Affiliations:** 1 Healthcare Services Department, Niğde Ömer Halisdemir University, Nigde, Turkey; 2 Histology-Embryology Department, Faculty of Medicine, Erciyes University, Kayseri, Turkey; 3 Histology-Embryology Department, Faculty of Medicine, Bozok University, Yozgat, Turkey; 4 Department of Medical Biology, Faculty of Medicine, Istinye University, Istanbul, Turkey; 5 Histology-Embryology Department, Faculty of Dentistry, Cappadocia University, Nevşehir, Turkey; 6 Gaziantep Islamic Science and Technology University, Department of Histology-Embryology, Gaziantep, Turkey

**Keywords:** Erythropoietin, Hypoxia-inducible factor-1, Methotrexate, Vascular endothelial growth – factor, Vitamin B12

## Abstract

**Objective(s):**

Methotrexate (MTX) is a drug with anti-inflammatory and immunosuppressive effects and is also a folic acid antagonist. Our aim in this study is to determine the molecular mechanisms of cardiotoxicity caused by MTX, a chemotherapeutic drug, and to evaluate the protective effects of vitamin B12 on this toxicity.

**Materials and Methods:**

A total of 32 rats were used in our study and 4 groups were formed. Control group, Vit B12 group (3 μg/kg B12 for 15 days, IP), MTX group (20 mg/kg MTX single dose on day 8 of the experiment, IP), MTX +Vit B12 group (3 μg/kg, IP ), Vit B12 throughout the 15 days, and a single dose of 20 mg/kg MTX (IP) on day 8 of the experiment. Immunohistochemically, expressions of hypoxia-inducible factor 1α (HIF1-α), vascular endothelial growth factor receptor-2 (VEGFR-2), erythropoietin (EPO), and interleukin-6 (IL-6) were evaluated in the heart tissue. Total catalase (CAT), superoxide dismutase (SOD), and malondialdehyde (MDA) levels were measured in the heart tissue. At the same time, ANP and NT-proBNP levels were measured in the blood serum.

**Results:**

In the study, the expression of HIF1-α and VEGFR-2 increased significantly in the MTX group, while IL-6 and EPO significantly decreased. At the same time, CAT and SOD levels were significantly decreased and MDA levels increased significantly in the MTX group. While vitamin B12 significantly corrected all these values, it also greatly reduced the increases in ANP and NT-proBNP levels caused by MTX.

**Conclusion:**

It is important to use Vit B12 before and after MTX administration to replace the folate that MTX has reduced.

## Introduction

Methotrexate (MTX) is a folate antimetabolite and cytotoxic chemotherapeutic agent used in the treatment of different malignancies such as head and neck tumors, osteosarcoma, and acute lymphoblastic leukemia (1). Despite its benefits in treating and possibly curing cancer, it is well known that chemotherapy can cause catastrophic damage to surrounding tissues, resulting in a range of potentially life-threatening cardiovascular toxicities and leading to serious health problems. These include numerous harmful side effects, including heart failure and myocardial infarction. Cardiotoxicity is responsible for significant long-term morbidity and mortality in patients, especially due to left ventricular dysfunction (2). Another application of MTX in low dosages is for the treatment of diverse autoimmune diseases, including rheumatoid arthritis (3-5). The frequent use of MTX in the treatment of both cancer and inflammatory diseases limits the applicability of the drug because it causes many side effects on other peripheral organs besides the target organs (6). 

MTX acts as an antagonist of folic acid, therefore it initially inhibits the enzyme dihydrofolate reductase, and then exerts its effect by inhibiting pyrimidines and purines required for RNA and DNA synthesis (7, 8). Therefore, MTX demonstrates cytotoxic effects not just on tumor cells, but also on vital organs, including the heart, by contributing to the toxicity of the drug with its inhibitory effect on glyoxalase and anti-oxidant systems (3). Cardiotoxicity refers to damage to the heart muscle and can occur during methotrexate treatment. This damage may be caused by methotrexate disrupting folate metabolism in heart muscle cells. Of all the organs, the heart is the most vulnerable to premature aging and oxidative stress caused by free radicals. Acute ischemia-reperfusion injury, hyperhomocysteinemia-induced endothelial damage, and chronic oxidative damage caused by lipid peroxidation can lead to an increase in free radicals. Although the heart is extremely susceptible and open to the effects of oxidative stress, it is also sensitive to the benefits of compounds such as anti-oxidants (8). Therefore in order to remove the detrimental effects of MTX as much as possible while preserving its therapeutic effect, using vitamins, drugs, or supplements that are known to be effective under these circumstances is significant (9). 

Vitamins are organic components that are indispensable for the normal metabolism of the human body but cannot be synthesized (9, 10). Considering that MTX is a folic acid antagonist, vitamin B must be used while MTX is administered. Water-soluble Vitamin B12 (Vit B12), which is acquired through diet, is associated with the synthesis and repair of DNA (11). It is also requisite for the production of tetrahydrofolate, which is important for DNA synthesis (12). Under normal circumstances, insufficient Vitamin B12 intake or deficiency could lead to chronic diseases such as cardiovascular disease, cancer, and osteoporosis (13). 

Oxidative stress, which is the basis of most diseases, starts at the level of cells and causes the cell to fail to preserve its structure and function, leading to dysfunction over time. One of these disorders may be in the form of dysfunction and impairment in the integrity of vascular endothelial cells. Hypoxia-induced factor-1alpha (HIF-1α) is one of the signs of drug toxicity in the cell. At present, a multitude of drugs are being clinically trialed as anticancer agents due to their capacity to impede angiogenesis (14). The increase or decrease in HIF-1α levels induces a corresponding increase or decrease in angiogenic factors, such as Vascular Endothelial Growth Factor (VEGF) and Erythropoietin (15), within the tissue. Therefore, the presentation of the existence of these molecules in cardiotoxicity is significant in ascertaining the toxicity caused by oxidative damage in the tissue. 

In addition, inflammatory markers such as IL-6, apart from natriuretic peptides, which are important markers of cardiac damage, are important for their use in the early detection of cardiotoxicity (16). 

In our study, natriuretic peptide levels and cytokine levels in the heart were specifically evaluated to detect organ damage in Mtx cardiotoxicity and to determine the protection and maintenance of structural integrity in the heart tissue. In light of these data, it is planned to reveal hypoxia-induced mechanisms and the response of heart tissue after oxidative stress by histological and biochemical methods by complementing the folate concentration reduced by MTX with Vit B12.

It aimed to examine many new aspects of MTX cardiotoxicity, such as HIF-1α, VEGFR-2, and EPO expressions, which were not analyzed in depth in previous studies. To show that these mechanisms change in the absence and presence of folate concentration, Vit B12 was used against the decreasing folate concentration in MTX cardiotoxicity.

This study aimed to elucidate the molecular mechanisms of cardiotoxicity that occur due to the anti-inflammatory and immunosuppressive effects of methotrexate and to evaluate the protective effects provided by Vit B12. Thus, we believe that it will significantly contribute to the limited literature on the cardiotoxic effect of methotrexate and will constitute a reference in this field.

## Materials and Methods

***Animals***

This study was designed and applied in the Department of Histology and Embryology, Erciyes University, Kayseri, Turkey. In this study, 32 male Wistar albino rats (10 weeks old, weighing 220-240 gr) were used. These rats received from Hakan Çetinsaya Experimental and Clinic Research Center, Erciyes University. The rats were housed in standard cages maintained at 21 ^°^C and subjected to a 12-hr light/dark cycle, with their nutritional and hydration needs met. The rats were weighed first. Animals of similar weight were brought together to form groups. All animals were maintained in accordance with established guidelines for their care and welfare.

***Experimental design***

MTX was obtained from Koçak Farma (Istanbul, Turkey). Vit B12 was obtained as 5,000 μg aliquots in glass tubes from Bristol-Myers Squibb (San Francisco, CA, USA). 

Control group (n=8): Throughout the experiment, the rats were administered saline intraperitoneally (IP). Vitamin B12 group (Vit B12)(n=8): During the entire study, the rats received a daily IP dose of 3 µg/kg B12 for 15 days, Methotrexate group (MTX)(n=8): On the eighth day of the study, the rats were administered a single IP dose of 20 mg/kg of MTX. Methotrexate+vitamin B12 group (MTX+Vit B12)(n=8): Throughout the 15-day study, the rats received a daily IP dose of 3 µg/kg of Vitamin B12, and on the eighth day, they were administered a single IP dose of 20 mg/kg of MTX (14). 

At the conclusion of the study, the animals were anesthetized with a mixture of 75 mg/kg of IP ketamine and 10 mg/kg of IP xylazine and subsequently euthanized. Their heart tissues were then extracted for histopathological and immunohistochemical examinations.

***Histological procedure***

Heart tissues were obtained from Rats. It was then placed in a fresh 10% formaldehyde solution. The heart tissues were rinsed with running tap water and subsequently subjected to a graded series of alcohols (50%, 70%, 80%, 96%, and three changes of absolute alcohol). After alcohol, the tissues were placed in xylene. After embedding in paraffin blocks, 5 µm-thick sections were cut into the slides. Both light microscopic and immunohistochemical stainings were performed on these sections.

***Hematoxylin and eosin (H*** ***&*** ***E) staining***

Paraffin was removed before heart tissue sections. It was then passed through xylene and alcohol solutions, respectively. Sections were first treated with a hematoxylin solution. Then washed in running water. Sections taken in eosin solution were rewashed after the procedure. They were then kept in alcohol and xylene.

***Immunohistochemistry procedure***

The staining was performed using the TA-125-HDX kit from Thermo Fisher Scientific. These were used as antibodies. Anti‐HIF‐1α (A16873; ABclonal), VEGF Receptor 2 (ab9530; Abcam), and Anti‐EPO (ab226956; Abcam). First, antigen retrieval was performed for the release of epitopes. Citrate buffer was employed for this purpose, followed by washing with phosphate-buffered saline (PBS). The manufacturer’s recommendations were taken into account in all procedures. Antibodies were detected using 3,3’-diaminobenzidine tetrahydrochloride (TA-060-HDX; Thermo Fisher Scientific). Counterstaining was performed with Gill hematoxylin. The sections were stained and subsequently photographed after they were dehydrated and sealed. The immunoreactivity intensities of the antibodies were measured by measuring 150 different fields from each group. ImageJ software was used for the measurement (17).

***Biochemical analyses***

*Elisa assay*

Heart tissues from rats stored at -80 ^°^C were homogenized before the study. Tissue samples were taken into empty tubes to obtain sera and were centrifuged at 1509 g for 10 min. After centrifugation, the supernatants were transferred to Eppendorf tubes for use. Superoxide dismutase (SOD), (Cat. No: 201-11-0169), Catalase (CAT), (Cat. No: 201-11-5106,), Malondialdehyde (MDA), (Cat. No: Cat. No: 201-11-0157), and Interleukin 6 (IL-6), (Cat. No: Cat. No: 201-11-0136) were measured in heart tissue samples. Atrial Peptide (ANP)(Cat. No: 201-11-0643) and N-terminal Natriuretic proB-natriuretic peptide (Cat. No: 201-11-0068) in blood serum samples was measured using the ELISA method. All kits were purchased from Sun Red Biological Technology Co., Ltd. (Shanghai, China), and were utilized according to the manufacturer’s recommended protocol. The measurement was carried out at 450 nm using an ELISA reader (17). 

***Statistical analyses***

Statistical analysis of this study was performed using GraphPad Prism version 8.00 for Mac (GraphPad Software, La Jolla, CA, USA). To determine whether the data were normally distributed, the D’Agostino-Pearson omnibus test was initially performed. If the data were normally distributed, differences between groups were determined using one-way analysis of variance (ANOVA) followed by the Tukey *post hoc* test. The data were presented as mean ± standard deviation of the mean for normally distributed data. For non-normally distributed data, the Kruskal-Wallis test was employed. To ascertain the differences between groups, Dunn’s multiple comparisons test was utilized. The results were expressed as the median (minimum-maximum), and a *P*-value less than 0.05 was regarded as statistically significant.

## Results

***Histopathological findings***

**Figure 1:**
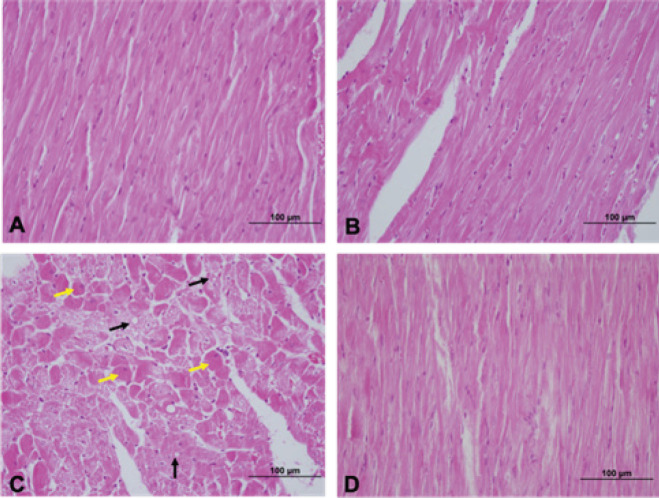
Hematoxylin and eosin staining of heart sections. Intracytoplasmic vacuoles (black arrow) and necrotic cells (yellow arrow) were shown

In the routine hematoxylin and eosin (H&E) stained histological sections, myocardial fibers and blood vessels exhibited normal histological appearances in both the control group and the group treated with vitamin B12. No evidence of abnormal histology was detected in the sections examined (Figures 1A and 1B). In the light microscopic examination, MTX group sections were observed to display disorganization between myocardial muscle fibers, eosinophilic staining in certain parts of the myocardial layer, increased development of intracytoplasmic vacuoles, and a necrotic appearance. In some MTX sections, congestive and hemorrhagic areas were observed in the vessels (Figure 1C). In the MTX+Vit B12 group, near-normal cardiac tissue appearance was observed in H&E staining and histopathological examination (Figure 1D).

***Immunohistochemical findings***

**Figure 2:**
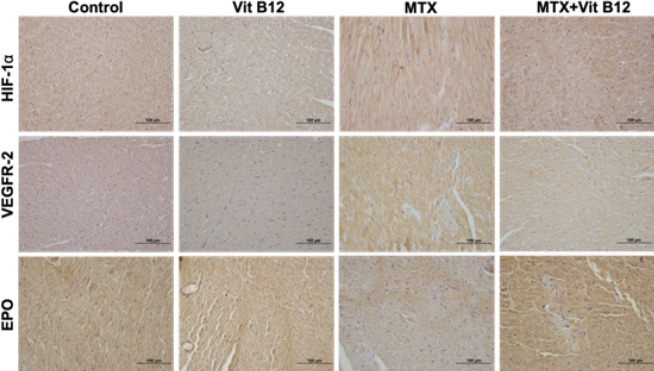
HIF-1α, VEGFR-2 and EPO expressions of all experimental groups of Wistar albino rats; Scale bar: 100 µm

MTX: methotrexate; Vit B12: vitamin B12; HIF-1α: hypoxia inducible factor-1alpha; VEGFR-2: vascular endothelial growth factor receptor-2; EPO: erythropoietin

While HIF-1α expression was similar between the control and vitamin B12 groups, a significant up-regulation was noted in the MTX group relative to the control group. The HIF-1α expression levels in the MTX+Vit B12 group were similar to those in the MTX group and were significantly higher than those observed in both Control and B12 groups (Figure 2). VEGFR-2 expression was similar in Control and Vit B12 groups; while a statistically significant increase was observed in the MTX group compared to the control and Vit B12 groups. There was a significant increase in the MTX+Vit B12 group compared to Control and Vit B12 groups and at the same time, a significant decrease was observed compared to the MTX group (Figure 2). When the expression of EPO was compared with the Control group and the MTX group, a significant decrease was observed in the MTX group. Upon comparison of the MTX group with the MTX+Vit B12 group, an important decrease in expression was observed in the MTX+Vit B12 group (Figure 2). HIF-1α, VEGFR-2, and EPO expressions of the groups are shown in Table 1. 

***Biochemical findings***

*Evaluation of oxidant/anti-oxidant system*

In the study, CAT, SOD, and MDA levels were examined in order to assess the oxidant/anti-oxidant system. “Regarding the CAT levels, a reduction was noted in the MTX group compared to the Control group, whereas an insignificant increase was observed in the Vit B12 and MTX+Vit B12 groups upon comparison with the Control group. Regarding the SOD level, when the Control group was compared with the other groups, there was a decrease in the MTX group and an increase in the Vit B12 and MTX+Vit B12 groups. However, these observed changes were not significant. Regarding the MDA level, when the Control group was compared with the other groups, a significant increase was observed in the MTX group, while the other groups were similar. CAT, SOD, and MDA levels of the groups are presented in Table 2.

*Evaluation of IL-6 level*

The IL-6 level was similar in the Control and Vit B12 groups. A decrease was observed in the MTX group compared to these groups. The IL-6 level that was decreased in the MTX group was increased in the MTX+Vit B12 group. These changes were not statistically significant. IL-6 levels of the groups are presented in Table 3.

*Evaluation of natriuretic peptides*

When the Control group was compared with the other groups, an elevation in ANP levels was noted in the MTX group, whereas a reduction was observed in the Vit B12 group. A statistically significant decrease was detected in the MTX+Vit B12 group compared to the Control group. Regarding the NT-proBNP level, when the Control group was compared with the other groups, an increase was observed in the MTX group, while a significant decrease was detected in the MTX+Vit B12 group. The Vit B12 group exhibited results similar to those of the Control group. ANP and NT-proBNP levels of the groups are shown in Table 4.

## Discussion

While cancer treatments carry the advantage of improving the survival of patients, they also bear side effects. The most common of these side effects is cardiovascular disease and there is a concern among cancer survivors that such effects may lead to early morbidity and death. The direct effects of cancer therapy on heart function and structure may be based on cardiotoxicity (18). MTX is acknowledged to develop reactive oxygen species (ROS) that leave oxidative damage in cancer cells and normal cells. The use of anti-oxidants during chemotherapy may decrease the formation of aldehydes from oxidative stress, which would result in improved treatment (19, 20). Following treatment with high doses of MTX, symptoms of cardiac toxicity including arrhythmias, hypotension, and cardiac arrest have been frequently reported (21, 22). Cardiomyopathy induced by chemotherapeutic agents is a major limiting factor for their application in cancer therapy. In a study evaluating the cardiotoxic effects on vascular endothelial cells and cardiomyocytes, the two main target cells of cardiovascular diseases, the cytotoxic effect of Mtx on endothelial cells could only be detected in the short term. (6) At low doses, MTX is absorbed orally by an active saturable transport mechanism, reaching peak concentrations within 1.5-2.5 hr after ingestion, and is eliminated primarily by renal excretion of unmetabolized MTX. At high doses, MTX has lower oral bioavailability and is metabolized by hepatic aldehyde oxidase to 7-hydroxy-MTX (increasing MTX-induced toxicity) (23). In some of the experimental studies, electrocardiographic changes of high doses of methotrexate were noted (24). These changes include atrial fibrillation, partial atrioventricular block, sinus arrhythmia sequences, and atrial flutter. These abnormalities have been associated with hypotension and, in some cases, death. In this animal model, electron microscopy revealed endothelial and platelet dysfunction that appeared to reflect direct cellular toxicity independent of the cytostatic effects of methotrexate. Additionally, these experimental studies show that the direct drug effects of methotrexate or the metabolic changes it causes may cause myocardial dysfunction (21). This indicates the need to conduct various studies evaluating agents with cardioprotective effects against MTX-induced damage. Therefore, our study aimed to evaluate cells damaged by inflammation, MTX cardiotoxicity, and possible hypoxia using biochemical and immunohistochemical methods and to examine the protective effects of compensation mechanisms using Vit B12 to increase blood circulation. In terms of MTX-induced cardiac damage and oxidative stress, numerous abnormalities were detected in the rat ventricle, such as mild hydrophobic changes in the striated myofibrillar structure, edema, myocardial atrophy, focal hemorrhage, cytoplasmic vacuoles, and nuclear pyknosis (25). Similarly, in some studies, a dosage of 20 mg/kg MTX was demonstrated to cause cardiac tissue destruction evidenced by histopathological changes in cardiac tissues (3, 25). In this study, in addition to the presence of necrotic cells in the heart tissues of the MTX group, irregularity in the myocardial muscle fiber, eosinophilia, and increased intracytoplasmic vacuoles in some parts of the myocardial layer indicate signs of cardiotoxicity due to the presence of congestive and hemorrhagic areas in some vessels. MTX exerts its impact by generating reactive oxygen species (ROS), leading to lipid peroxidation and consequent impairment of mitochondrial function (26). The decrease in CAT and SOD levels, as well as the increase in MDA level, could be argued to be a proof of this effect. In our study, it was determined that Vit-B12 along with MTX applied for 15 days improved CAT, SOD, and MDA both histologically and biochemically, and therefore regulated the cardiotoxicity caused by MTX by acting on folate. The body responds to changes in oxygen levels by modifying the rate of respiration and blood flow at the organism level. On a cellular level, alterations in oxygen levels can prompt a range of signaling pathways to induce changes in patterns of gene expression (14). HIF-1α is one of the general factors of transcription-changing cellular responses to hypoxia which plays a significant role in the initiation or regulation of inflammation (27, 28). It is also a crucial transcription factor for cardiovascular improvement and systemic oxygen homeostasis. In hypoxic conditions or due to genetic alterations, the aberrant and excessive expression of HIF-1α plays a crucial role in cancer biology, as well as several other pathophysiological issues such as vascularization, angiogenesis, cell survival, other diseases, or oxidative stress (29). The key regulator of oxygen homeostasis, HIF-1, has been reported to increase during myocardial hypoxia, which impacts the importance of mitochondrial function since it regulates oxygen delivery and utilization as well as redox homeostasis (30-32). In this study, an increase in HIF-1α was observed in the MTX group, which is consistent with the deterioration in histology and biochemical parameters. Tissue hypoxia, which is observed to be a normal physiological response to reduced tissue perfusion induces HIF-1α activity, activating the transcription of genes encoding angiogenic factors (31). VEGF is one of the primary genes involved in the angiogenesis cascade in myocardial ischemia, regulated by HIF. The induction and control of angiogenesis, with VEGFR-2 increasing in parallel with the increase in HIF-1α, is affected in correlation with the interaction of different cardiac cellular hypoxic and inflammatory disorders (30, 33). Regarding HIF-1α and VEGFR-2, hypoxia is a factor related to inflammation and angiogenesis, thus, their expression can be argued as an early indicator of myocardial hypoxia (33). Physiological levels of VEGFR-2 are required to preserve normal endothelial function, while it should be underlined that very high concentrations can lead to pathological changes, including apoptosis (28). Low tissue oxygen tension due to HIF-1α activation also leads to EPO gene expression through both transcriptional activation and mRNA stabilization (34). In addition to its contribution to the production of red blood cells, EPO is regarded as a tissue-protective cytokine and is known to have angiogenic, anti-apoptotic, anti-oxidative, and anti-inflammatory effects (34-36). In this study, a decrease in EPO expression was observed in contrast to the increased expression of HIF-1α and VEGFR-2 in the MTX group. It is known that EPO and its receptor are expressed not only in tissues related to erythropoiesis but also in a number of different organs such as the heart (35). Considering that EPO is secreted mainly by the kidneys in response to hypoxia, it would exist in lesser amounts in other organs compared to the kidney. Therefore, it could be argued that the amount of EPO may have decreased due to the decrease in the amount of EPO due to the low amount of EPO in the heart or the decrease in the available EPO receptor after MTX damage. It is considered that Vit B12 corrects the folate deficiency caused by MTX in cardiomyocytes and regulates hypoxia-induced cellular responses. However, while Vit B12 did not cause a change in the MTX-induced increased HIF-1α expression in heart tissue, a significant decrease was observed in the expression of VEGFR-2 and EPO. We believe that extending the duration of vitamin B12 or increasing its dose will correct hypoxia by reducing HIF-1α expression. 

In this study, the presence of IL-6, which is one of the key inflammatory cytokines that play a role in cardiac remodeling, was also investigated. IL-6 is a cytokine that is essential in the initiation and progression of atherosclerotic lesions. Acute infection, chronic inflammatory disease, obesity, and physiological stress cause the production of IL-6. Increased concentration of IL-6 has many effects, including propagation of the inflammatory response, production of acute phase reactants, and increased coagulability (16). Several studies in the literature have shown that MTX-induced hepatic, renal, and cardiac damage in mice is linked to a marked elevation in IL-6 levels (37, 38). However, contrary to these studies, in this study a decrease in tissue IL-6 levels was found. IL-6 is known to be both a pro-inflammatory cytokine and an anti-inflammatory myokine released from skeletal muscles (39). In our study, it can be thought that MTX, as an anti-inflammatory drug, suppressed IL-6 levels in heart tissue. Because, based on the folate antagonistic effect of MTX, inflammatory cell function is regulated and low-dose (5-25 mg/week) MTX leads to an anti-inflammatory effect in diseases such as rheumatoid arthritis (40, 41). In this study, it was observed that the IL-6 level in the Vit B12 administered group presented values close to the control group and the group that received only Vit B12. The 20 mg/kg MTX dosage we used in our study may have reduced the IL-6 level in the heart tissue due to its anti-inflammatory effects.

In various studies, ANP and NT-proBNP levels were investigated by investigating the endocrine function as well as the contractile function of the heart (42, 43). NT-proBNP is a biomarker of both systolic and diastolic heart insufficiency (44). In this study, an increase was observed in both ANP and NT-proBNP levels in the MTX group. Based on MTX cardiotoxicity, it can be thought that this increase is secreted from cardiomyocytes. A decrease in these values was observed in the group administered Vit B12 together with MTX, and it was seen that Vit B12 could regulate cardiomyocyte dysfunction in the tissue by reducing the cardiotoxic effects of MTX.

(A) Control group, (B) Vit B12 group, (C) MTX group, and (D) MTX+Vit B12 group. Scale bar: 100 µm

MTX: methotrexate; Vit B12: Vitamin B12

**Table 1:** Expression measurement results of hypoxia-inducible factor 1α (HIF1-α), vascular endothelial growth factor receptor-2 (VEGFR-2) and erythropoietin (EPO) immunohistochemical stainings in heart tissues of all experimental groups

**Groups**	**Control**	**Vit B12**	**MTX**	**MTX+Vit B12**	***p***
**HIF-1α ** expression	159.418 (142.9-177.8)^a^	160.777 (138.8-179.9)^a^	170.185 (145.5-180.7)^b^	170.701 (144.5-171.1)^b^	0.0001
**VEGFR-2 ** expression	164.765 (142.9-184.2)^a^	164.498 (137.3-180.2)^a^	180.048 (157.3-189.0)^b^	174.120 (150.8-187.9)^c^	0.0001
**EPO** expression	165.755 (125.5-177.0)^a^	163.409 (148.4-177.7)^ab^	161.345 (139.2-179.0)^b^	156.029 (106.4-176.4)^c^	0.0001

Data are expressed as median (min-max). There was no significant difference between groups containing the same letter (a and b). *P<*0.05 was considered significant

MTX: methotrexate; Vit B12: Vitamin B12

**Table 2:** ELISA results of catalase (CAT), superoxide dismutase (SOD), and malondialdehyde (MDA) levels are given in heart tissue samples

**Groups**	**Control**	**Vit B12**	**MTX**	**MTX+Vit B12**	***p***
**CAT levels**	107.5±14.7^ab^	112±16.8^a^	75.35±11.2^b^	114.1±26.5^a^	0.012
**SOD levels**	23.99±7.6^a^	24.2±9.2^a^	18.58±8.6^a^	32.44±7.2^a^	0.10
**MDA levels**	9.3±3.1^a^	9.5±1.4^a^	14.2±1.4^b^	9.8±1.3^a^	0.0036

Data are expressed as mean±standard deviation. There was no significant difference between groups containing the same letter (a and b). *P<*0.05 was considered significant

MTX: methotrexate; Vit B12: Vitamin B12

**Table 3:** The ELISA results relating to IL-6 levels in the heart tissue samples of every experimental groups are presented

**Groups**	**Control**	**Vit B12**	**MTX**	**MTX+Vit B12**	***p***
**IL-6 levels**	89.9±12.5	89.2±18.8	28.2±12.6	93.4±12.2	0.53

Data are expressed as mean±standard deviation. *P<*0.05 was considered significant. There is no statistically significant difference between the groups

MTX: methotrexate; Vit B12: Vitamin B12

**Table 4:** Atrial Peptide (ANP) and NT-proBNP levels in blood serum in all groups

**Groups**	**Control**	**Vit B12**	**MTX**	**MTX+Vit B12**	***p***
**ANP levels**	220.2±44.0^a^	212.3±27.7^a^	263.7±27.7^a^	145.4±39.8^b^	0.0002
**NT-proBNP levels**	257.2±45.3^a^	261.2±43.6^a^	307.9±74.8^a^	155.5±24.4^b^	0.0004

Data are expressed as mean±standard deviation. There was no significant difference between groups containing the same letter (a and b). *P<*0.05 was considered significant

MTX: methotrexate; Vit B12: Vitamin B12

## Conclusion

In this study, folate deficiency was created using MTX and this deficiency was tried to be compensated with Vit B12 supplementation. Activation of HIF-1α with hypoxia that develops after oxidative stress caused by MTX was demonstrated in addition to changes in VEGFR-2 and EPO expressions that develop. Since vascular homeostasis is known to be a balanced state between endothelial cells in the vascular wall and vascular smooth muscle cells, regulation of VEGFR-2, an important receptor for the bioactive substance VEGF, which is necessary to maintain this balance, constitutes the basic principle of the study. In this study, the authors suggested the efficacy of Vit B12 in MTX-induced hypoxia by stimulating angiogenesis through the up-regulation of VEGF rather than EPO, which is not primarily secreted in the heart tissue. Thus, the authors suggest that impaired CAT, SOD, and MDA values in MTX cardiotoxicity, histopathological change in cardiomyocytes, and endocrine activity of the heart (ANP and NT-proBNP levels) can be repaired by Vit B12.

This study suggested the effectiveness of Vit B12 in MTX-induced hypoxia by stimulating angiogenesis through up-regulation of VEGF. Thus, it is thought that CAT, SOD, and MDA values, histopathological changes in cardiomyocytes, and endocrine activity of the heart (ANP and NT-proBNP levels) damaged in MTX cardiotoxicity can be repaired with Vit B12.

## Authors’ Contributions

N K, D K, and E K contributed to the development of the idea or hypothesis generation, as well as the planning methodology for the research and article. N K and E K were involved in the procurement process of the materials needed for the experimental phase of the project. N K, D K, E K, AT A, T C, AB Y, and B Y actively participated in conducting the experiments, ensuring effective follow-up, performing data analysis, and preparing the report. Moreover, all authors reviewed the intellectual content and checked for spelling errors before finalizing the article.

## Funding

This work was financially supported by the Scientific Research Projects Unit of Erciyes University, Türkiye (TSA-2019-8673 project code).

## Ethics Committee Approval

All procedures were carried out with the approval of the Ethical Committee (decision No:18/116 and subject heart tissue 22/097, date 2018) of Experimental Animals of Erciyes University.

## Conflicts of Interest

The authors declare that there are no conflicts of interest.
